# Long‐term exposure to non‐steroidal anti‐inflammatory (NSAID) medication in relation to dementia risk: a population‐based cohort study

**DOI:** 10.1002/alz.088721

**Published:** 2025-01-09

**Authors:** Ilse vom Hofe, Bruno H. Stricker, M. Kamran Ikram, Frank J. Wolters, M. Arfan Ikram

**Affiliations:** ^1^ Erasmus University Medical Center, Rotterdam Netherlands; ^2^ Erasmus University Medical Center, Rotterdam, Zuid‐Holland Netherlands

## Abstract

**Background:**

Non‐steroidal anti‐inflammatory (NSAID) medication are suggested to have beneficial effects in the prevention of Alzheimer’s disease, due to anti‐inflammatory and possibly amyloid‐lowering properties. However, the results of observational studies and randomized‐controlled trials have been inconsistent, and duration and dose‐response relationships are still unclear.

**Method:**

We included 11,745 dementia‐free participants from the prospective population‐based Rotterdam Study (59.5% women, mean age 66.2 years). NSAID use from 1991 was derived from pharmacy dispensing records, from which we determined cumulative duration and dose. We defined four mutually exclusive categories of cumulative use: non‐use, short‐term use (<1 month), intermediate‐term use (between 1‐24 months), and long‐term use (>24 months). We determined the association with dementia risk until 2020 using Cox regression models, including NSAID use as time‐varying exposure. Models were adjusted for lifestyle factors, comorbidity and comedication use. We repeated the analyses stratified by previously established amyloid‐lowering properties of different NSAIDs.

**Result:**

During an average follow‐up period of 14.5 years, a total of 9,520 (81.1%) participants had used NSAIDs at any given time, and 2,091 participants developed dementia. Use of NSAIDs was associated with lower dementia risk for long‐term users (hazard ratio (HR) [95%CI]: 0.88[0.84;0.91]), but not with short‐term use (HR[95%CI]: 1.04[1.02;1.07]) or intermediate term use (HR[95%CI]: 1.04[1.02;1.06]). The cumulative dose of NSAIDs was not associated with decreased dementia risk (HR[95%CI] for <25^th^ percentile: 1.06[1.03;1.09], 25‐50^th^ percentile: 1.02[0.99;1.05], 51‐75^th^ percentile: 1.03[0.99;1.06], >75^th^ percentile: 0.99[0.96;1.02]). Associations were somewhat stronger for NSAIDs without known effects on amyloid than for amyloid‐lowering NSAIDs (HR[95%CI]: 0.79[0.74;0.85] versus 0.89[0.85;0.93]).

**Conclusion:**

Long‐term NSAID use, but not cumulative dose, was associated with decreased dementia risk. This suggests that prolonged rather than intensive exposure to anti‐inflammatory medication may hold potential for dementia prevention.

